# Circulating prosaposin and ependymin-related protein 1 levels are correlated with insulin resistance in type 2 diabetic patients

**DOI:** 10.3389/fendo.2025.1519586

**Published:** 2025-06-26

**Authors:** Xin Ji, Yajing Wang, Sijia Xu, Ling Cao, Penghua Fang, Zhenwen Zhang

**Affiliations:** ^1^ Department of Endocrinology, Northern Jiangsu People’s Hospital Affiliated to Yangzhou University, Yangzhou, China; ^2^ Department of Endocrinology, Northern Jiangsu People’s Hospital, Yangzhou, China; ^3^ Key Laboratory for Metabolic Diseases in Chinese Medicine, First College of Clinical Medicine, Nanjing University of Chinese Medicine, Nanjing, China

**Keywords:** PSAP, EPDR1, T2DM, insulin resistance, adipokine

## Abstract

**Objective:**

Skeletal muscle and adipose tissues secrete myokines and adipokines to regulate energy metabolism. Experimental evidence indicates that prosaposin (PSAP) and ependymin-related protein 1 (EPDR1) are involved in the regulation of thermogenesis and energy metabolism. To our knowledge, little literature has been found dealing with PSAP and EPDR1 levels in type 2 diabetes mellitus (T2DM) patients. The aim of the study was to evaluate possible relationships between both peptide levels and insulin resistance indexes in type 2 diabetic subjects.

**Methods:**

The study groups consisted of 64 T2DM subjects and 22 normal controls. Serum PSAP and EPDR1 concentrations were determined using immunosorbent assay kits.

**Results:**

The serum PSAP (319.6 ± 78.38 vs. 207.2 ± 42.43, P<0.0001) and EPDR1 (7.988 ± 3.484 vs. 6.399 ± 3.788, P=0.0823) concentrations were higher in T2DM subjects than normal controls. In addition, positive correlations were found between: PSAP and fasting blood glucose (FBG) levels (r = 0.5004; P< 0.0001), PSAP and Hemoglobin A1c (HAb1c) (r = 0.4688; P< 0.0001), PSAP and C-peptide (r = 0.3981; P = 0.0003), PSAP and triglyceride-glucose index (TyG) (r = 0.2362; P< 0.0001), PSAP and homeostasis model assessment of insulin resistance (HOMA-IR) (r = 0.3314; P = 0.0035), PSAP and homeostasis model assessment of C-peptide resistance (HOMA-CR) (r = 0.5486; P< 0.0001), EPDR1 and insulin (r = 0.2291; P = 0.045), EPDR1 and HOMA-IR (r = 0.2462; P = 0.0309) in both T2DM and normal control subjects.

**Conclusions:**

Our results indicated that T2DM individuals have higher serum PSAP and EPDR1 levels, and both peptide concentrations were positively correlative to insulin (INS) resistance levels. PSAP and EPDR1 levels may be taken as potential biomarkers to forecast the development of T2DM.

## 1 Introduction

One of the major health issues facing the globe today is the increasing rise of diabetes. The IDF Diabetes Atlas (10th edition) estimated that 10.5% (536.6 million) of adults in the world aged 20 to 79 would have diabetes in 2021 and 12.2% (783.2 million) in 2045 (1). It should be noted that T2DM, which is caused by the combination of various pathogenic factors, is primarily caused by insulin resistance (2). Therefore, understanding the precise molecular mechanism by which pathogenic variables cause insulin resistance will facilitate the development of innovative therapies to combat the condition.

More recently, the idea that released cytokines from adipose tissue and skeletal muscle play a significant role in the pathogenesis of insulin resistance and T2DM has received a great deal of favorable attention (3). In this regard, PSAP and EPDR1 have been recently described as myokines and adipokines that are involved in the regulation of fat browning and energy metabolism in animals (4, 5). The central and peripheral nervous systems both contain large amounts of PSAP, a precursor to the lysosomal proteins Saposins A–D, which have neurotrophic effects (6, 7). Additionally, a prior study revealed that both adipose tissues and muscle express and secrete PSAP (4). The adipogenic gene program of adipocytes and the genes involved in oxidative phosphorylation are both affected by the forced expression of PASP in primary inguinal fat cells (4), although its precise involvement in insulin resistance is yet unknown.

EPDR1 was recently identified as a secreted adipokine regulating mitochondrial respiration linked to thermogenesis in brown fat (5). Firstly, human islets from T2DM and obese donors had elevated EPDR1 mRNA expression, which was positively linked with the donors’ BMI (8). In contrast, it was discovered that in the subcutaneous adipose tissue of healthy obese participants, EPDR1 was downregulated after both short- and long-term weight loss (9). Additionally, long-term insulin treatment significantly increases endothelial EPDR1 expression (10), which in turn encourages cell migration and the development of capillary tubules. An increasing body of research also suggests that EPDR1 can control the body’s whole energy metabolism by regulating the metabolism of target cells’ mitochondria and improving β-cell function (4, 5, 8).

To our knowledge, however, very little research has been conducted on the specific topic of serum PSAP and EPDR1 concentrations in T2DM patients. Therefore, we conducted this cross-sectional study to assess the levels of the peptides PSAP and EPDR1 in the blood of individuals with T2DM, as well as investigate any potential associations between these peptide levels and markers of insulin resistance among the subjects.

## 2 Materials and methods

The present study was conducted at the Northern Jiangsu People’s Hospital Affiliated to Yangzhou University. The present study consisted of 64 T2DM volunteers and 22 healthy volunteers with a normal weight according to a physical examination and routine laboratory tests. In this study, each participant gave official written consent, had normal exercise and eating behavior, and had no fat preference or fat aversion. Individuals with active hepatitis or liver cirrhosis, hypertension, chronic renal failure on hemodialysis, congestive heart failure, or other known major diseases were precluded from the study. The assignment of patients with T2DM and normal controls must meet the diagnostic criteria of the World Health Organization (11). For T2DM: fasting venous serum value: ≥126 mg/dl or 75 g (2-h) oral glucose tolerance test (OGTT) venous serum value: ≥200 mg/dl. Patients without typical symptoms need to be retested on different days. For normal controls: fasting venous serum value: <100 mg/dl or 75 g (2-h) OGTT venous serum value: <140 mg/dl. The protocol of this study was approved by the Ethics Committee of Northern Jiangsu People’s Hospital Affiliated to Yangzhou University (No. 2023ky200).

For each case, body mass index (BMI) was calculated at the time of blood collection as weight in kilograms divided by height in meters squared. The HOMA-IR Index was calculated for each participant using the formula [fasting glucose (mmol/L) × fasting insulin (mIU/L)/22.5]. The HOMA-β (INS) index was calculated as [20 × fasting insulin (mIU/L)/(fasting glucose (mmol/L)-3.5) (%)]. The homeostasis model assessment of insulin sensitivity (HOMA-IS) index was calculated using the formula [1/HOMA-IR]. The HOMA-CR index was calculated as [1.5+fasting glucose (mmol/L) × fasting C peptide (ng/ml)/(2.8×0.333)]. The homeostasis model assessment of beta-cell function (HOMA-β) (C-peptide) index was calculated as [270×fasting C-peptide (ng/ml)/(fasting glucose (mmol/L)-3.5) (%)]. After an overnight fast, blood samples were collected from each participant at 8:00 a.m. and immediately centrifuged (4°C) as described previously (9). Briefly, within 30 minutes of collection, the blood samples (2 mL) were placed in prechilled EDTA tubes containing 100 μl of aprotinin (1 μg/mL) and centrifuged for 15 min. at 1000 g at 4°C. The separated serum was placed into vials and kept at -80°C until it was measured. A radioimmunoassay was used to measure the levels of serum insulin. Regular biochemical tests were run on the Olympus AU2700 automatic chemistry analyzer, including glucose, triglyceride (TG), total cholesterol (TC), high-density (HDL-C), and low-density lipoprotein cholesterol (LDL-C). The leftover serum was immediately kept at -80°C for the purpose of determining PSAP and EPDR1 following standard analysis.

An enzyme-linked immunosorbent test (Cloud-Clone, Inc., Wuhan, China) was used to examine the serum levels of human PSAP (SEC756Hu) and EPDR1 (SEQ610Hu). According to the manufacturer’s specification, the assay range for PSAP was 0.78–50 ng/mL, and the average sensitivity was 0.33 ng/mL, intra-assay precision CV%<10%, and inter-assay precision CV%<12%, as well as the assay range for EPDR1 was 0.156–10 ng/mL, and the average sensitivity was 0.054 ng/mL, intra-assay precision CV%<10%, and inter-assay precision CV%<12%. The mean of the two measurements, which were all taken in duplicate, was taken into account.

## 3 Statistical analysis

The statistical analyses were performed with GraphPad Prism v6.0 (GraphPad Software). All data were presented as mean ± SD. The Kolmogorov-Smirnov test was used to assess the normality of the data. The differences between the groups were analyzed with an independent t-test or a Mann-Whitney test in the non-parametric distributions (not normally distributed). Possible correlations between parameters were evaluated by Pearson’s correlation coefficient analyses (normally distributed) or Spearman’s correlation coefficient analyses (not normally distributed). Statistical significance was considered to be P< 0.05.

## 4 Results

### 4.1 Serum PSAP and EPDR1 levels were increased in T2DM individuals

**Figure 1:**
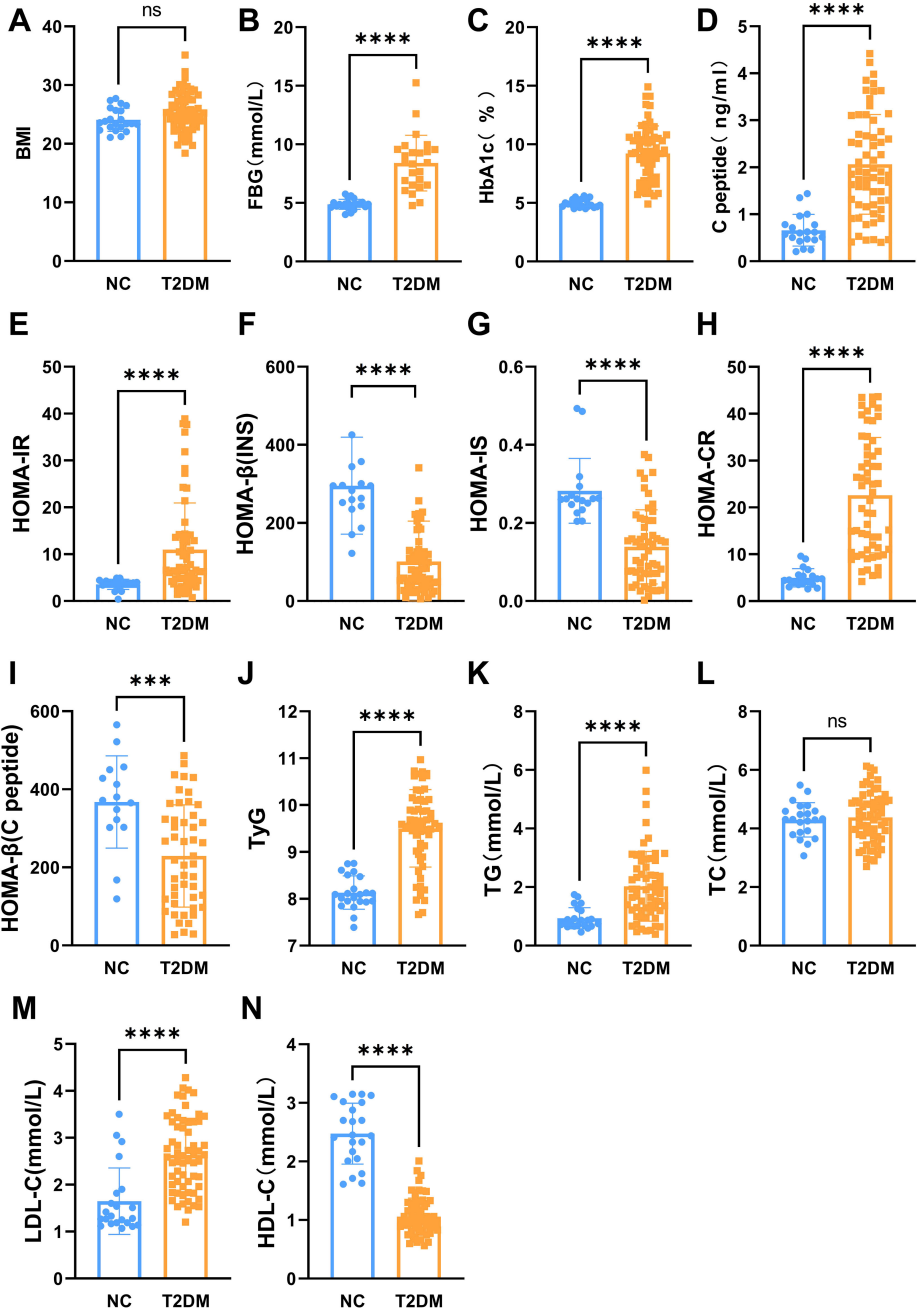
The main biochemical characteristics of two groups. The BMI **(A)**, fasting blood glucose **(B)**, HbA1c **(C)**, C-peptide **(D)**, HOMA-IR **(E)**, HOMA-CR **(H)**, TyG **(J)**, TG **(K)**, and LDL-C **(M)** levels were significantly increased in patients with T2DM. The HOMA-b (INS) **(F)**, HOMA-IS **(G)**, HOMA-b (C-peptide) **(I)**, and HDL-C **(N)** were decreased in patients with T2DM. No significant differences in TC **(L)** levels in patients with T2DM. Data are expressed as mean ± SD, ****P < 0.0001; ***P < 0.001; ns, no significance.

**Figure 2:**
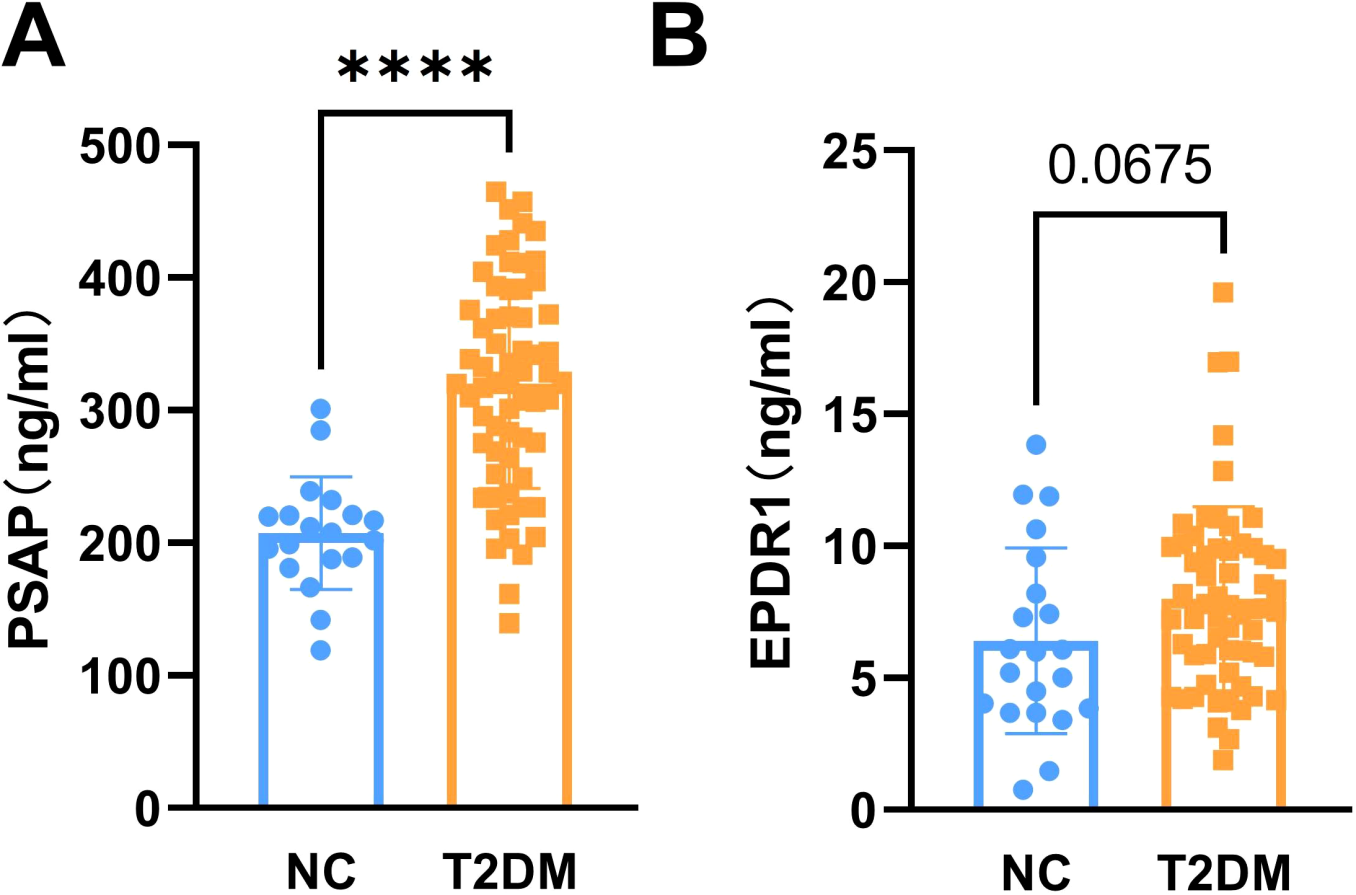
The levels of serum PSAP and EPDR1 in T2DM subjects. The serum PSAP **(A)** and EPDR1 **(B)** concentrations were increased in T2DM subjects. Data are expressed as mean ± SD, ****P<0.0001 vs. normal controls.

The main indexes of age, duration of diabetes, body height, body weight, SBP, DBP, and insulin in the T2DM group and the normal control group are listed in **Table 1**. The results showed that fasting blood glucose (FBG), HbA1c, C-peptide, HOMA-IR, HOMA-CR, TyG, TG, and LDL-C levels were significantly higher in patients with T2DM than normal controls (**Figure 1**). Besides, HOMA-β (INS), HOMA-β (C-peptide), HOMA-IS, and HDL-C were decreased in patients with T2DM compared with the normal controls (**Figure 1**). There were no significant differences in insulin or TC levels in patients with T2DM compared with the normal controls (**Figure 1**, **Table 1**). Interestingly, the serum PSAP (319.6 ± 78.38 vs. 207.2 ± 42.43, P<0.0001) and EPDR1 (7.988 ± 3.484 vs. 6.399 ± 3.788, P=0.0823) concentrations were significantly higher in T2DM subjects compared to normal control controls (see **Figure 2**). The diabetic history and medication use of the NC group and T2DM group are listed in **Table 2**.

**Table 1:** Biochemical and demographic characteristics of two groups.

Characteristics	NC	T2DM	p value
N	22 (male, 8; female 14)	64 (male, 38; female 26)	
Age (years)	47.95 ± 6.90	49.48 ± 13.20	0.491
Duration of diabetes (years)	0	5.5 (5,10)	<0.001
Body height (cm)	167.86 ± 6.889	167.05 ± 8.20	0.676
Body weight (kg)	67.82 ± 6.19	71.06 ± 12.54	0.118
SBP (mmHg)	114.68 ± 13.89	135.06 ± 18.39	<0.001
DBP (mmHg)	70.14 ± 10.59	79.33 ± 11.50	<0.001
Fasting Insulin (uIU/mL)	17.89 (16.59,19.15)	17.43 (12.33,29.67)	0.114

Results are shown as means ± SD, [M(P25, P75)]; N, number of cases; Statistical significance p<0.05.

**Table 2:** Diabetic history and medication use of the NC group and T2DM group.

Characteristics, n (%)	NC (n=22)	T2DM (n=64)
Smoking history		
Never	2 (9.09)	5 (7.81)
Former	1 (4.55)	3 (4.69)
Current	1 (4.55)	1 (1.56)
Hypertension	2 (9.09)	31 (48.44)
Hyperlipidemia	1 (4.55)	44 (68.75)
Non-alcoholic fatty liver disease	1 (4.55)	22 (34.38)
Diabetic complications		
Type 2 diabetes mellitus microvascular complications		18 (28.13)
Type 2 diabetes mellitus macrovascular complications		28 (43.75)
Type 2 diabetes mellitus peripheral neuropathy		10 (15.63)
Medication use at baseline		
Insulin and analogues		22 (34.38)
Insulin sensitizers (Biguanides, TZDs)		31 (48.44)
Insulin secretagogues (Sulfonylureas, Glinides)		15 (23.44)
Incretin modulators (GLP-1RA, DPP-4i)		13 (20.31)
Renal glucose excretion drugs (SGLT-2i)		9 (14.06)
α-Glycosidase inhibitors		6 (9.38)

### 4.2 Serum PSAP and EPDR1 were positively correlative to insulin resistance in T2DM individuals

**Figure 3:**
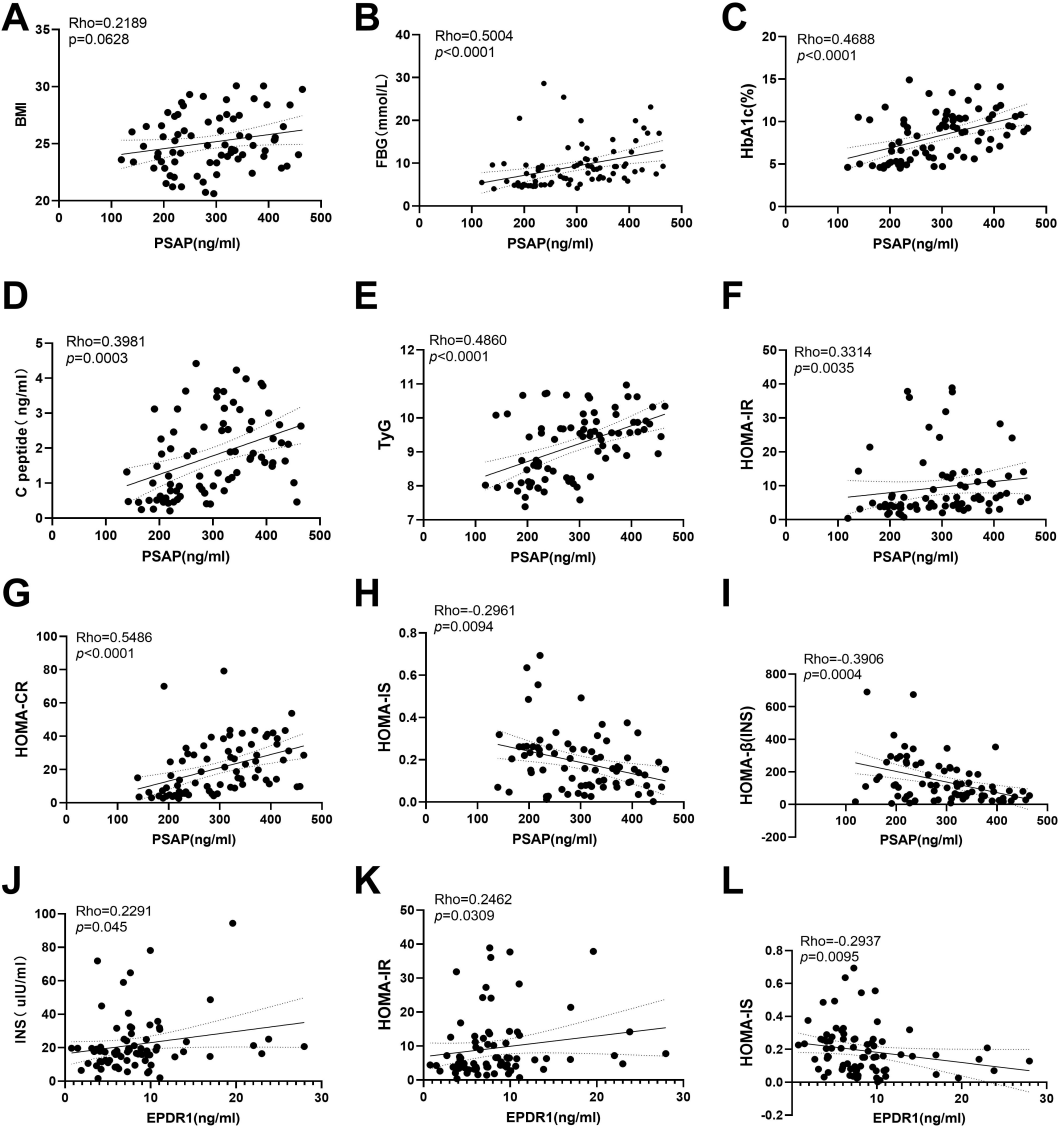
Correlations between serum PSAP and EPDR1 and insulin resistance indexes. No significant correlations were found between PSAP levels and BMI **(A)**. The positive correlations were found between PSAP and Fasting blood glucose **(B)**, PSAP and HAb1c **(C)**, PSAP and C-peptide **(D)**, PSAP and TyG **(E)**, PSAP and HOMA-IR **(F)**, PSAP and HOMA-CR **(G)**, EPDR1 and insulin **(J)**, EPDR1 and HOMA-IR **(K)** in both T2DM and normal control groups. The negative correlations were found between PSAP and HOMA-IS **(H)**, PSAP and HOMA-β (INS) **(I)**, and EPDR1 and HOMA-IS **(L)**.

To investigate whether serum PSAP and EPDR1 levels were associated with insulin resistance, we performed analysis of the correlation between PSAP and EPDR1 and indicators of FBG levels (r = 0.5004; P< 0.0001), PSAP and HAb1c (r = 0.4688; P< 0.0001), PSAP and C-peptide (r = 0.3981; P = 0.0003), PSAP and TyG (r = 0.2362; P< 0.0001), PSAP and HOMA-IR (r = 0.3314; P = 0.0035), PSAP and HOMA-CR (r = 0.5486; P< 0.0001), EPDR1 and insulin (r = 0.2291; P = 0.045), EPDR1 and HOMA-IR (r = 0.2462; P = 0.0309) in both T2DM and normal control groups. In contrast, negative correlations were found between PSAP and HOMA-IS (r = -0.2961; P = 0.0094), PSAP and HOMA-β (INS) (r = -0.3906; P = 0.0004), and EPDR1 and HOMA-IS (r = -0.2937; P = 0.0095). However, no significant correlations were found between PSAP levels and BMI (r = 0.2189; P = 0.0626) (**Figure 3**).

## 5 Discussion

Obesity is considered to be a promoting factor of T2DM, which is usually accompanied by T2DM and glucose and lipid metabolism disorders, based on the same pathophysiological basis. It is well known that adipocytes secrete a large number of factors involved in the process of regulating energy metabolism in the body. Growing data suggests that PSAP and EPDR1 are involved in animal energy metabolism and glucose homeostasis (4, 5, 8). It should be noted that PSAP expression and secretion are associated with the remodeling of thermogenic fat brought on by cold and are necessary for the expression of thermogenic genes and oxidative phosphorylation (4). Additionally, PSAP has been connected to lysosome and mitochondrial organellar interactions (12). Furthermore, forced PSAP expression was sufficient to increase oxygen consumption rates in primary inguinal fat cells (4), while PSAP knockdown using siRNA in murine bone marrow-derived macrophages lowered oxygen consumption rates (13). To date, the expression and secretion of PSAP in T2DM have not yet been studied. This study found that patients with T2DM had significantly higher serum PSAP levels. It’s interesting to note that PSAP and insulin resistance indices like FBG, HAb1c, C-peptide, TyG, HOMA-IR, and HOMA-CR showed positive relationships. Increased insulin resistance in T2DM patients may be a possible signal promoting PSAP production and release. Consequently, the higher PSAP level may be used as a novel biomarker to anticipate the rise in insulin resistance linked to T2DM.

EPDR1, another adipokine, is important in the emergence of obesity and metabolic disorders (4, 5, 8). However, to our knowledge, little literature has been reported on EPDR1 levels in T2DM. Our study revealed that circulating EPDR1 levels were higher in T2DM patients compared to healthy controls, which is consistent with the upregulation of EPDR1 in brown fat and pancreatic islets in obese individuals (5, 8). Hyperglycemia or hyperinsulinemia associated with T2DM may be a possible reason for the observed elevated EPDR1 levels. First, it has recently been discovered that EPDR1 is a target gene for non-canonical insulin signaling in endothelial cells (10). Second, there was a clear negative link with Hb1Ac and a clear linear positive correlation between EPDR1 mRNA levels and insulin release in human islets from obese patients (8). Third, EPDR1 silencing in human islets and β-cell lines decreased glucose-stimulated insulin secretion (8), whereas treatment with human EPDR1 protein increased glucose-stimulated insulin secretion (8), indicating that upregulating EPDR1 in obese individuals may improve β-cell function to maintain glucose homeostasis. Additionally, we discovered a significant positive association between serum EPDR1 and insulin levels, which is consistent with this explanation.

Notably, T2DM-related insulin resistance may be another explanation for the observed elevated EPDR1 levels. Previous research has shown that the elevation of EPDR1 in human islets from obese individuals is a result of an adaptive strategy to deal with an increased metabolic demand brought on by insulin resistance by improving glucose responsiveness (8). We also discovered a high positive association between serum EPDR1 and HOMA-IR, which supports this hypothesis. Contrary to what we expected, the PSAP, EPDR1, and BMI levels of the T2DM participants in this investigation did not significantly correlate with one another. Future research should focus on the differences in the impact of peptides on BMI in humans and animals and whether diabetes drugs improve IR through these two peptides. The fact that the simple size evaluated was not sufficient is another weakness in this study. In order to confirm the reliability of these findings, it is necessary to continue to collect simple observations in the future.

## 6 Conclusion

Taken together, blood levels of EPDR1 and PSAP were considerably greater in T2DM patients compared to healthy controls and were correlated with enhanced insulin resistance levels. Therefore, increased serum PSAP and EPDR1 levels in T2DM participants suggest increased blood insulin resistance levels. Similar to this, increased PSAP and EPDR1 levels in the serum may be predicted by higher degrees of insulin resistance. The levels of PSAP and EPDR1 may be used as possible biomarkers to predict the emergence of insulin resistance and T2DM.

## Data Availability

The original contributions presented in the study are included in the article/supplementary material. Further inquiries can be directed to the corresponding authors.
